# Soft Robotics: Academic Insights and Perspectives Through Bibliometric Analysis

**DOI:** 10.1089/soro.2017.0135

**Published:** 2018-06-01

**Authors:** Guanjun Bao, Hui Fang, Lingfeng Chen, Yuehua Wan, Fang Xu, Qinghua Yang, Libin Zhang

**Affiliations:** ^1^ College of Mechanical Engineering, Zhejing University of Technology, Hangzhou, China.; ^2^ Library, Zhejiang University of Technology, Hangzhou, China.

**Keywords:** soft robotics, artificial muscle, bioinspired robot, smart material, multidisciplinary, bibliometrics

## Abstract

Soft robotics is of growing interest in the robot community as well as in public media, and there is an increase in the quality and quantity of publications related to this topic. To formally elaborate this growth, we have used a bibliometric analysis to evaluate the publications in the field from 1990 to 2017 based on the Science Citation Index Expanded database. We present a detailed overview and discussion based on keywords, citation, h-index, year, journal, institution, country, author, and review articles. The results show that the United States takes the leading position in this research field, followed by China and Italy. Harvard University has the most publications, high average number of citations per publication and the highest h-index. *IEEE Transactions on Robotics* ranks first among the top 20 academic journals publishing articles related to this field, whereas *Soft Robotics* holds the top position in journals categorized with “ROBOTICS.” Actuator, fabrication, control, material, sensing, simulation, bionics, stiffness, modeling, power, motion, and application are the hot topics of soft robotics. Smart materials, bionics, morphological computation, and embodiment control are expected to contribute to this field in the future. Application and commercialization appear to be the initial driving force and final goal for soft robots.

## Introduction

In recent years, soft robotics has become one of the fastest growing topics in the robotic community, and its rise in academia^1^ suggests the potential to revolutionize the role of robotics in society and industry.^2^ Despite this prodigious future, the research field is quite young. According to a survey of the literature, the term “soft robot” was first used for a rigid pneumatic hand,^3^ which had a certain degree of object compliance owing to the compressibility of gas. Afterward, soft robot was gradually used in a variety of articles, patents, reports, and other scientific documents, yet still represented a robot or similar machine composed of rigid materials. In 2008, the term “soft robotics” was adopted to describe investigation of rigid robots with compliant joints,^4^ as well as soft material-based robots with large scale flexibility, deformability, and adaptability.^5^

But the efforts to invent new robots that are totally different from their conventional rigid counterparts really started far before the appearance of the professional terminology. In the 1950s, McKibben developed braided pneumatic actuators for an orthotic appliance for polio patients.^6^ The McKibben artificial muscle was widely investigated and employed in different types of robot design.^7^ In 1990, Shimachi and Matumoto^8^ reported their work on soft fingers. One year later, Suzumori et al.^9^ published their flexible microactuator made of silicone rubber and tried several applications.^10–12^ In the following dozen years, similar structures were developed, named as pneumatic bellows actuators,^13^ electrostrictive polymer artificial muscle actuators,^14^ rubbertuator,^15^ fluidic muscle,^16^ pneumatic rotary soft actuator,^17^ flexible fluidic actuator,^18^ flexible pneumatic actuator,^19^ tentacle manipulator,^20^ elephant trunk manipulator,^21^ Air-Octor,^22^ OctArm,^23^ caterpillar robot,^24^ Clobot,^25^ continuum manipulators,^26^ and so on. Despite the different mechanisms, structures, and motion performance, these actuators and devices are clearly key developments in the discipline of soft robotics.^27,28^

Although soft robotics has a history of almost half a century, it has only become a hot topic in the science community and the general public in the most recent decade. As these technologies are gradually recognized by the robotic community, more and more scientists and engineers look to contribute to the field. This is reflected by the ever-increasing number of laboratories, international collaborations, emerging publications, soft robot-related societies and organizations, special session in all kinds of international conferences, professional events, and activities. Although the soft robotics field is still in its infancy, a number of review articles have been published to summarize the achievements, analyze the techniques, and discuss the challenges and prospects for the future. These reviews were organized in terms of technical contents but we would like to present a different perspective by using bibliometric analysis to show the historical map and overall view of the soft robotics research field.

Bibliometric analysis is quite effective for analyzing scientific publications to map the historical development of the target topic, find the hotspots, highlight the distribution layout of active researching countries, institutions, authors, and their cooperation relations, as well as top journals for publications, leading influence articles, and research trends. It has been adopted in a variety of disciplines, such as chemistry,^29^ economics,^30^ computing,^31^ management,^32,33^ education,^34^ medicine,^35^ energy,^36,37^ and robotics.^38^ Yet, to our knowledge, this is the first bibliometric analysis to assess the soft robotics research field. Our goal is to provide a general overview on this research area by revealing the following aspects: (1) historical map of the topic; (2) the main contributors: countries, institutes, research groups, authors, and leading research areas; (3) cooperation patterns between countries, institutes, and authors; (4) the most productive journals; (5) top articles with highest citation number; and (6) research interests and perspectives.

## Methodology and Data Source

The analysis is based on the publications related to “soft robot” published from 1985 to 2017. Literature were retrieved through the Science Citation Index-Expanded and Social Science Citation Index on August 17, 2017, with search formula of “Artificial muscle*” or “Pneumatic muscle* actuat*” or “continuous robot*” or “redundant robot*” or “soft robot*” or “soft wearable robot*” or “Bio inspired Robot*” or “Bioinspired Robot*” or “soft bodied robot*” or “bio soft robot*” or “biomimetic* robot*”or “biological* inspire* robot*” or biorobotic* or microrobotic* or “bio robot*” or bioactuat* or “redundant actuat*,” defining the document type as article and review in the field of topic. As a result, 1495 articles were collected from InCites data set including Web of Science (WOS) content indexed through May 31, 2017. Articles originated from England, Scotland, Northern Ireland, and Wales were grouped under the United Kingdom heading. Keyword and international cooperation were analyzed by Thomson Data Analyzer. The impact factor (IF) for each journal was determined according to the report of 2016 Journal Citation Reports. Since the WOS “topic” searching was applied to the title, abstract, and keyword fields defining the document type as article and review, some other related publications may not be covered.

## Results and Discussions

### Global contribution and leading countries

Although literature retrieving covers the time span from 1985 to 2017, articles concerning soft robots were first published in 1990. From then on, 70 countries have contributed to the soft robotics research field with 1495 publications, in which 37 are Essential Science Indicators (ESI) high cited articles and 4 are ESI hot articles.

**FIG. 1.**
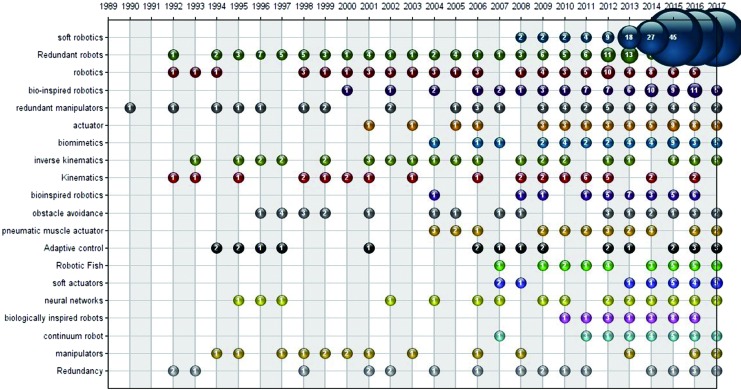
Bubble chart of top 20 author keywords by year. Color images available online at www.liebertpub.com/soro

**FIG. 2.**
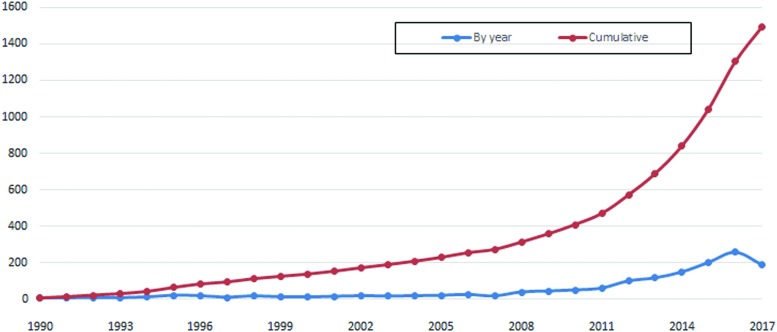
Trends in the number of published articles related to soft robotics by year. Color images available online at www.liebertpub.com/soro

The term “soft robotics” was initially used to represent rigid robot that had compliant joints and variable stiffness.^4^ Then soft robots were distinguished from traditional robots and soft robotics became a new multidisciplinary field involving soft material-based structure with compliance and deformability in the interaction with environment.^5^ From 2008, “soft robotics” was widely adopted as a keyword in scientific articles, especially after 2012, shown in Figure 1. The emerging trend of publications with the keyword “soft robotics” is consistent with the polyline shown in Figure 2. Although the first articles related to soft robotics emerged in 1990, the number of total publications per year was relatively stable ranging from 7 to 27 during 1990–2007, suggesting that this domain was not so attractive to scientists and engineers at that time.

But just 1 year later (2008), at the beginning of OCTOPUS IP,^39^ the Large-Scale Integrating Project funded by the European Commission under the 7th Framework Programme, we can see publications increased to 41. The trend continued with increments of several articles until 2012, when the number of publications rose 66% to 101 articles on a year-on-year basis. Since then, the rising rate of yearly publications has been relatively stable, with high percentages of 17%, 30%, 35%, and 27% in 2013, 2014, 2015, and 2016, respectively. The active interests and intensive efforts from research and engineering communities in the past few years has led to the enormous growth of this field, which is supported by a large increase in the number of publications.^40^ The total number of articles published in the most recent 5 years is more than three times of that on this topic in the first 18 years since 1990. In the first 5 months of 2017, there already exist 191 articles in the soft robotics field, which will certainly contribute to another bigger increase this year than the 260 articles in 2016.

Table 1 shows the top 20 countries in terms of the number of publications related to the field of soft robotics. The United States is the most productive country with a total of 478 articles since 1990, followed by China (230 articles) and Italy (149 articles). Although we cannot attribute this productivity to particular causes, these countries are the focus of several new funding programs such as the DARPA ChemBots program in the United States in 2008, the major national research funding initiative Tri-Co Robot in China from 2016, and the OCTOPUS IP at the BioRobotics Institute of Scuola Superiore Sant'Anna^41^ in Italy. The BioRobotics Institute is also the primary host of a Coordination Action for Soft Robotics funded by the European Commission under the Future and Emerging Technologies—FET-Open Scheme that hosted a series of activities and events.^40^

**Table 1.** The Top 20 Most Productive Countries in Soft Robotics Field During 1990–2017

*Rank*	*Country*	*TA*	*TC*	*ACPP*	*SP (%)*	*nCC*
1	United States	478	10811	22.62	28.66	34
2	China	230	1771	7.7	30.87	18
3	Italy	149	2226	14.94	42.95	26
4	South Korea	83	1123	13.53	28.92	12
5	United Kingdom	81	1220	15.06	53.09	25
6	Germany	80	1183	14.79	65	29
7	France	70	994	14.2	40	17
8	Japan	69	1219	17.67	43.48	17
9	Canada	53	494	9.32	47.17	13
10	Switzerland	45	918	20.4	53.33	18
11	Australia	37	346	9.35	48.65	11
12	Singapore	37	451	12.19	54.05	14
13	New Zealand	30	258	8.6	46.67	13
14	Spain	25	157	6.28	44	9
15	Israel	23	213	9.26	30.43	5
16	India	22	212	9.64	54.55	10
17	Turkey	20	179	8.95	35	4
18	Iran	20	102	5.1	35	6
19	The Netherlands	19	450	23.68	78.95	15
20	Greece	18	202	11.22	44.44	5

TA, total articles; TC, total citations; ACPP, average citations per publication; SP, share of publications; nCC, number of cooperative countries.

Of the top 20 country's publications, a large number (>28%) are international articles, especially for the Netherlands (78.95%) and Germany (65%). This implies that soft robotics has attracted worldwide scientists and engineers to exchange ideas and cooperate with each other. Another observation is that despite the high number of publications from China (second with 230 articles), the average citations per publication (ACPP) is relatively low, only 7.7%. It is unclear whether this reflects a language barrier, a bias in accessing different publications, or the scope and quality of the research itself.

**FIG. 3.**
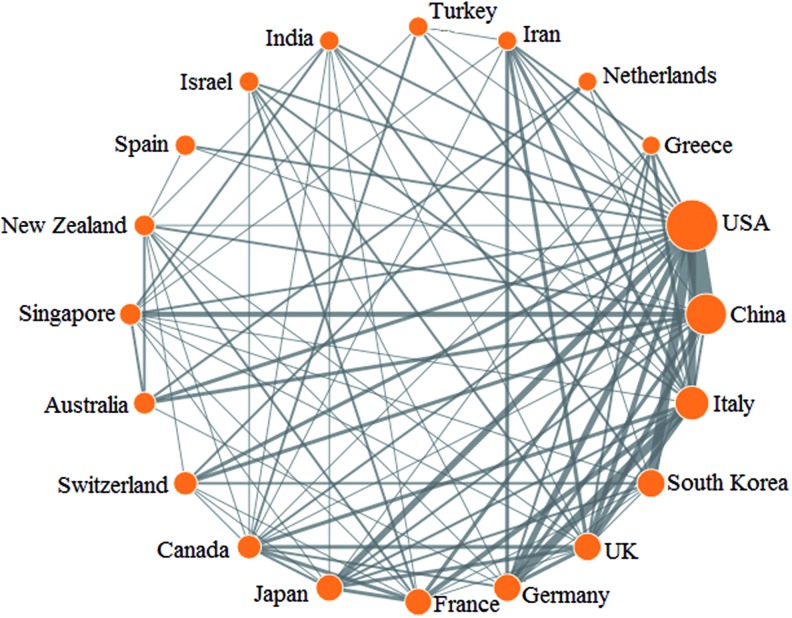
Collaboration matrix map among the top 20 productive countries. Color images available online at www.liebertpub.com/soro

Figure 3 shows the collaborative relationship of the top 20 productive countries. The size of nodes is proportional to the total number of articles of each country. The lines represent collaboration between countries, the thickness of which indicates the intensity of cooperation. The United States is the most active country that collaborated with 50 countries, especially with China, Italy, Germany, South Korea, and Japan. Germany lists on the second place, followed by Italy and the United Kingdom. One of the possible reasons might be the Europe visa policy that makes the European research institutes easy to recruit researchers from other countries within Europe. Another reason relies on the European research council that provides lots of cooperation opportunities for researchers from different European countries.

### Contribution of leading institutions

Table 2 shows the top 20 productive institutions in soft robotics research along with their total number of publications, citations, and h-index. Apparently, most of them are from top 10 productive countries. Harvard University leads the list with the most publications followed by Scuola Superiore Sant'Anna and Chinese Academy of Sciences. As for the ACPP, Harvard University and Massachusetts Institute of Technology (MIT) lead the list with 43.12 and 40.76, respectively. The two universities also have the highest h-index as 27 and 20. Clearly these institutions have taken a prominent role in developing and promoting the field. Furthermore, another four institutions from the United States given in Table 2 also have relatively high ACPP values, namely Carnegie Mellon University (14.57), University of California System (21.67), University of Michigan (25.25), and Cornell University (22.13). However, the four Chinese institutions in the top 20 list have relatively low ACPP results, <10. Compared with the influence of the American and Italian research institutions, the Chinese counterparts need more efforts to improve their research work and global influence in this field to match with its position in publication list.

**Table 2.** The Top 20 Most Productive Institutions of Publications During 1990–2017

*Rank*	*Institutions*	*TA*	*TPR%*	*TC*	*ACPP*	*h-Index*	*Country*
1	Harvard University	65	4.35	2803	43.12	27	United States
2	Scuola Superiore Sant'Anna	48	3.21	906	18.88	13	Italy
3	Chinese Academy of Sciences	43	2.88	300	6.98	9	China
4	Massachusetts Institute of Technology	42	2.81	1712	40.76	20	United States
5	Istituto Italiano di Tecnologia	40	2.68	692	17.3	12	Italy
6	Carnegie Mellon University	37	2.47	539	14.57	14	United States
7	University of California System	33	2.21	715	21.67	14	United States
8	Centre National de la Recherche Scientifique	32	2.14	425	13.28	10	France
9	Sun Yat Sen University	29	1.94	281	9.69	11	China
10	University of Auckland	25	1.67	192	7.68	8	New Zealand
11	Seoul National University	24	1.61	319	13.29	9	South Korea
12	National University of Singapore	20	1.34	107	5.35	6	Singapore
13	Beihang University	19	1.27	118	6.21	6	China
14	Ecole Polytechnique Federale de Lausanne	19	1.27	353	18.58	8	Switzerland
15	University of Wollongong	19	1.27	163	8.58	7	Australia
16	Tsinghua University	17	1.14	143	8.41	5	China
17	Swiss Federal Institute of Technology Zurich	17	1.14	188	11.06	8	Switzerland
18	University of Michigan	16	1.07	404	25.25	9	United States
19	Cornell University	16	1.07	354	22.13	9	United States
20	Nanyang Technological University	16	1.07	368	23	8	Singapore

TPR%, the percentage of articles of journals in total publications.

### Contribution of leading research areas

As is well known, soft robotics is a quite new developing multidisciplinary field,^42^ which is also supported by articles distributed in 91 WOS research areas. Table 3 illustrates the top 20 WOS research areas ranked by the number of articles related to soft robotics. There is no doubt that “Robotics” dominates the research area list with 557 articles, followed by “Automation and Control Systems,” “Engineering, Electrical and Electronic,” “Engineering, Mechanical,” “Materials Science, Multidisciplinary,” and “Computer Science, Artificial Intelligence,” which are the main scientific areas that put special emphasis on soft robotics. “Physics, Condensed Matter,” “Chemistry, Multidisciplinary,” and “Chemistry, Physical” lead the list of ACPP, with ACPP of 27.33, 25.75, and 24.73, respectively. These three areas are closely related with the current hot topic in soft robotics: smart material, even biohybrid material. The high ACPP in these areas verifies the widely accepted opinion that material is the key for soft robot development.^2,43–45^

**Table 3.** Contribution of the Top 20 Research Areas in Soft Robotics Field

*Rank*	*WOS research area*	*TA*	*TPR%*	*TC*	*ACPP*
1	Robotics	557	37.26	8777	15.76
2	Automation and Control Systems	262	17.53	4662	17.79
3	Engineering, Electrical, and Electronic	205	13.71	3951	19.27
4	Engineering, Mechanical	183	12.24	1996	10.91
5	Materials Science, Multidisciplinary	167	11.17	3214	19.25
6	Computer Science, Artificial Intelligence	163	10.90	2098	12.87
7	Instruments and Instrumentation	117	7.83	1309	11.19
8	Engineering, Multidisciplinary	99	6.62	1137	11.48
9	Engineering, Manufacturing	95	6.35	1418	14.93
10	Nanoscience and Nanotechnology	95	6.35	1997	21.02
11	Physics, Applied	94	6.29	1680	17.87
12	Materials Science, Biomaterials	92	6.15	1207	13.12
13	Chemistry, Multidisciplinary	73	4.88	1880	25.75
14	Chemistry, Physical	59	3.95	1459	24.73
15	Mechanics	48	3.21	483	10.06
16	Engineering, Biomedical	42	2.81	328	7.81
17	Computer Science, Interdisciplinary Applications	41	2.74	278	6.78
18	Physics, Condensed Matter	40	2.68	1093	27.33
19	Computer Science, Theory and Methods	29	1.94	313	10.79
20	Computer Science, Cybernetics	26	1.74	516	19.85

WOS, Web of Science.

### Leading journals in terms of number of publications in soft robotics research

The 1495 articles related to soft robotics during 1990–2017 were published in 357 journals. As listed in Table 4, *IEEE Transactions on Robotics* takes the leading position with 64 articles, followed by *Bioinspiration & Biomimetics* (62), *IEEE-ASME Transactions on Mechatronics,* (57) and *Soft Robotics* (53). The aforementioned four journals share 15.79% of the total publications, and the top 20 journals listed in Table 4 have produced 656 articles with the share of 43.88%. And all the remaining journals contribute with shares less than 1% each. In terms of IF, *Soft Robotics* has the highest value of 8.649 except the two material-related journals *Advanced Materials* and *Advanced Functional Materials.* Because of its overwhelming high IF, *Soft Robotics* holds the top position in journals categorized with “ROBOTICS” in the latest two consecutive years since its first IF indexed by WOS in 2015, as show in Table 5.

**Table 4.** The Top 20 Journals Publishing Articles in Soft Robotics Field

*Rank*	*Journal title*	*TA*	*TC*	*ACP*	*IF*
1	*IEEE Transactions on Robotics*	64	1481	85.94	4.036
2	*Bioinspiration & Biomimetics*	62	1002	80.65	2.939
3	*IEEE-ASME Transactions on Mechatronics*	57	1211	73.68	4.357
4	*Soft Robotics*	53	474	60.38	8.649
5	*International Journal of Robotics Research*	44	1414	97.73	5.301
6	*Robotica*	42	315	78.57	1.554
7	*International Journal of Advanced Robotic Systems*	39	87	58.97	0.987
8	*Robotics and Autonomous Systems*	35	566	82.86	1.95
9	*Journal of Intelligent & Robotic Systems*	32	422	75	1.512
10	*Advanced Robotics*	31	319	90.32	0.92
11	*Mechanism and Machine Theory*	27	362	85.19	2.577
12	*Smart Materials and Structures*	26	73	65.38	2.909
13	*Journal of Robotic Systems*	21	321	95.24	n/a
14	*IEEE Transactions on Robotics and Automation*	19	1004	100	n/a
15	*Advanced Materials*	19	488	78.95	19.791
16	*Advanced Functional Materials*	17	551	88.24	12.124
17	*Journal of Mechanisms and Robotics-Transactions of the ASME*	17	49	70.59	2.371
18	*IEEE Transactions on Industrial Electronics*	17	288	82.35	7.168
19	*Industrial Robot-An International Journal*	17	100	58.82	0.863
20	*Sensors and Actuators A-Physical*	17	185	81.25	2.499

IF, impact factor; ACP, article cited percentage.

**Table 5.** The Top Five Journals Categorized in “ROBOTICS” by Web of Science

*JCR year*	*Rank*	*Full journal title*	*TC*	*IF*
2015	1	*Soft Robotics*	150	6.130
	2	*Bioinspiration & Biomimetics*	1,285	2.891
	3	*Swarm Intelligence*	339	2.577
	4	*International Journal of Robotics Research*	4,590	2.489
	5	*Robotics and Computer-Integrated Manufacturing*	1,931	2.077
2016	1	*Soft Robotics*	356	8.649
	2	*International Journal of Robotics Research*	8,754	5.301
	3	*Journal of Field Robotics*	2,267	4.882
	4	*IEEE Transactions on Robotics*	12,478	4.036
	5	*IEEE Robotics & Automation Magazine*	2,383	3.276

JCR, Journal Citation Reports.

**FIG. 4.**
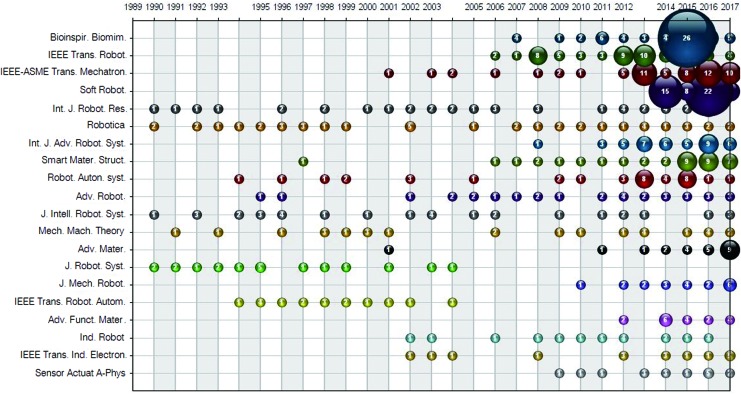
Bubble chart of top 20 productivity journals by year. Color images available online at www.liebertpub.com/soro

To show the historical map of soft robotics-related publications in journals, we employ the bubble chart of top 20 productivity journals by year, shown in Figure 4. It can be seen that there were few articles sparsely distributed in the top 20 journals from 1990 to 2008. After a 4-year significant increase, the soft robotics field witnessed an explosive growth in publications from 2012 and this is a continuing growing trend. This pattern agrees with that shown in Figure 2. Apart from robotics-oriented journals, the top journals contributing to this increasing trend in soft robotics are in the fields of materials science or bionics such as *Smart Materials and Structures*, *Advanced Materials*, *Advanced Functional Materials,* and *Bioinspiration & Biomimetics*.

Another journal, *Science Robotics* launched at the end of 2016,^46^ is not included in this analysis. However, in the published 9 issues, 13 out of 41 total articles are soft robotics related. The 31.71% of soft robot-focused articles indicate the deep interest and close attention of the journal in this field.

### Contribution of leading authors

Table 6 shows the top 10 most productive authors based on the number of publications. Whitesides group leads the list with the total number of publications of 18 followed by Wood (13), Laschi (12), Cianchetti (12), Yu (12), and Walsh (12). For the ACPP, Whitesides group ranks the first with number of 69.33, followed by Wood (34.85), and Laschi (32.25). Whitesides group also achieves the highest h-index of 14, followed by Wood (11) and Laschi (6). Interestingly, Prof. Whitesides laboratory is based in a Chemistry Department that enables him to develop soft robots from the viewpoint of chemistry and materials.^45^ Table 7 illustrates the top five authors with high total citation, all of whom are from Whitesides research group. All these top 10 productive authors are from the top 3 productive countries, indicating that there are certain concentrating groups in this field, such as the Whitesides research group at Harvard University and the Octopus research group in Europe (mainly at Scuola Superiore Sant'Anna, Italy). Nevertheless, the share of articles of the top 10 authors in total publications is only 7.88%, which means that a large number of researchers are working in this field and make contributions in the total 1495 publications. The vast population in this research community will certainly yield extraordinary progress and diverse achievements in the near future.

**Table 6.** Contribution of the Top 10 Authors in Soft Robotics Research

*Rank*	*Author*	*TA*	*TPR%*	*TC*	*ACPP*	*h-Index*	*Institution*
1	Whitesides	18	1.2	1248	69.33	14	Harvard University
2	Wood	13	0.87	453	34.85	11	Harvard University
3	Laschi	12	0.8	387	32.25	6	Scuola Superiore Sant'Anna
4	Cianchetti	12	0.8	202	16.83	4	Scuola Superiore Sant'Anna
5	Yu	12	0.8	75	6.25	3	Chinese Academy of Sciences
6	Walsh	12	0.8	222	18.5	6	Harvard University
7	Tan	11	0.74	131	11.91	6	Chinese Academy of Sciences
8	Rus	10	0.67	453	45.3	7	Massachusetts Institute of Technology
9	Mazzolai	9	0.6	163	18.11	4	Istituto Italiano di Tecnologia
10	Cho	9	0.6	214	23.78	6	Seoul National University

**Table 7.** Top Five Authors with High Total Citation

*Rank*	*Author*	*TA*	*TPR%*	*TC*	*ACPP*	*h-Index*	*Institution*
1	Whitesides	18	1.20	1248	69.33	14	Harvard University
2	Shepherd	9	0.60	731	81.22	9	Harvard University
3	Chen	3	0.20	700	233.33	3	Harvard University
4	Mazzeo	3	0.20	624	208	3	Harvard University
5	Ilievski	3	0.20	623	207.67	3	Harvard University

### Analysis of the most cited articles

Although the citation impact of an article will be influenced by many factors,^47^ it is still a widely accepted index for evaluating scientific articles. We list the top 10 most cited publications for analysis, shown in Table 8. The most highly cited article is “Self-organization, embodiment, and biologically inspired robotics” published in *Science* by Pfeifer *et al.*^48^ It leads the list of total citations (356). Whereas “Multigait soft robot”^49^ and “An electrically and mechanically self-healing composite with pressure and flexion-sensitive properties for electronic skin applications”^50^ take the second and third places according to their total citations (353 and 330) with quite high annual citations (50.4 and 55), respectively. Moreover, it should be noted that the recently published article “Design, fabrication and control of soft robots” (2015)^42^ in *Nature* gains the highest total citation per year (69), which to some extent reveals the wide concern on this field.

**Table 8.** Top 10 Most Cited Publications During the Period of 1990–2017

*No.*	*Author*	*Title*	*TC*	*TCY*	*Source*	*Year*	*Country*
1	Pfeifer *et al.*	Self-organization, embodiment, and biologically inspired robotics	356	32.4	Science	2007	Switzerland
2	Shepherd *et al.*	Multigait soft robot	353	50.4	Proceedings of the National Academy of Sciences of United States of America	2011	United States
3	Tee *et al.*	An electrically and mechanically self-healing composite with pressure- and flexion-sensitive properties for electronic skin applications	330	55	Nature Nanotechnology	2012	United States
4	Ilievski *et al.*	Soft robotics for chemists	306	43.7	Angewandte Chemie-International Edition	2011	United States
5	Suo	Theory of dielectric elastomers	269	33.6	Acta Mechanica Solida Sinica	2010	China
6	Kim *et al.*	Soft robotics: a bioinspired evolution in robotics	235	47	Trends in Biotechnology	2013	United States and Italy
7	Chen *et al.*	Modeling of biomimetic robotic fish propelled by an ionic polymer-metal composite caudal fin	227	28.4	IEEE-ASME Transactions on Mechatronics	2010	United States
8	Kim *et al.*	Smooth vertical surface climbing with directional adhesion	213	21.3	IEEE Transactions on Robotics	2008	United States
9	Rus and Tolley	Design, fabrication and control of soft robots	207	69	Nature	2015	United States
10	Kim *et al.*	Stretchable nanoparticle conductors with self-organized conductive pathways	201	40.2	Nature	2013	United States and South Korea

TCY, total citations per year.

Among these top 10 articles, 6 were published in top journals such as *Science*,^48^ *Nature,*^42,50,51^ and IEEE transactions.^52,53^ Extraordinarily, eight are from institutes in the United States (two have coauthors from institutes in Italy and South Korea), indicating that the United States is the leading country in this research field. The other two articles are authored by Swiss^48^ and Chinese researchers,^54^ respectively. Again, the Whitesides group shows its position and influence in soft robotics by contributing 2 articles^45,49^ among the 10 most highly cited publications. Moreover, “Design, fabrication and control of soft robots” (2015)^42^ holds the first position of the ESI hot article, followed by “Stretchable, skin-mountable, and wearable strain sensors and their potential applications: a review” (2016),^55^ “Soft robotic glove for combined assistance and at-home rehabilitation,” (2015)^56^ and “Phototactic guidance of a tissue-engineered soft-robotic ray” (2016).^57^

### Research interests and perspectives

As listed in Table 9, the first review on soft robotics was published in 1999.^58^ There was no other review for a decade before soft robotics attracted wide interests in 2008. After that, there were reviews on soft robotics each year. Especially after 2014, there were at least four reviews on this topic, which again implies that soft robotics is a hot spot in the robotic community. The multidisciplinary nature of soft robotics and the variety of authors' professional backgrounds result in a diversity of contents, analysis perspectives, and arguments in their publications. Despite the different perspectives, all the reviews listed in Table 9 were conceived from the technical standpoints, such as actuator, fabrication, control, material, sensing, simulation, bionics, stiffness, modeling, power, motion, and application, as shown in Table 10.

**Table 9.** Reviews on Topic of Soft Robotics

*Year (No.)*	*Author*	*Title*	*Source*	*Reference*
1999	Robinson and Davies	Continuum robots—a state of the art	*Proceedings of the 1999 IEEE ICRA*	^58^
2008 (1)	Trivedi *et al.*	Soft robotics: biological inspiration, state of the art, and future research	*Applied Bionics & Biomechanics*	^59^
2008 (2)	Zhang *et al.*	Review on flexible pneumatic actuator and its application in dexterous hand	*China Mechanical Engineering*	^70^
2009 (1)	Cho *et al.*	Review of manufacturing processes for soft biomimetic robots	*International Journal of Precision Engineering & Manufacturing*	^71^
2009 (2)	Greef *et al.*	Toward flexible medical instruments: Review of flexible fluidic actuators	*Precision Engineering*	^72^
2010	De Volder and Reynaerts	Pneumatic and hydraulic microactuators: a review	*Journal of Micromechanics & Microengineering,*	^73^
2011 (1)	Iida and Laschi	Soft robotics: challenges and perspectives	*Procedia Computer Science*	^44^
2011 (2)	Ilievski *et al.*	Soft robotics for chemists	*Angewandte Chemie-International Edition*	^45^
2012 (1)	Pfeifer *et al.*	The challenges ahead for bio-inspired soft robotics	*Communications of the ACM*	^62^
2012 (2)	Yujun *et al.*	Review of soft-bodied robots	*Journal of Mechanical Engineering*	^74^
2012 (3)	Otero *et al.*	Biomimetic electrochemistry from conducting polymers. A review: Artificial muscles, smart membranes, smart drug delivery and computer/neuron interfaces	*Electrochimica Acta*	^75^
2013	Kim *et al.*	Soft robotics: a bio-inspired evolution in robotics	*Trends in Biotechnology*	^64^
2014 (1)	Majidi	Soft robotics: a perspective—current trends and prospects for the future	*Soft Robotics*	^2^
2014 (2)	Laschi and Cianchetti	Soft robotics: new perspectives for robot bodyware and control	*Frontiers in Bioengineering & Biotechnology*	^1^
2014 (3)	Bauer *et al.*	A soft future: from robots and sensor skin to energy harvesters	*Advanced Materials*	^65^
2014 (4)	Bahramzadeh and Shahinpoor	A review of ionic polymeric soft actuators and sensors	*Soft Robotics*	^63^
2015 (1)	Elango and Faudzi	A review article: investigations on soft materials for soft robot manipulations	*International Journal of Advanced Manufacturing Technology*	^76^
2015 (2)	Rus and Tolley	Design, fabrication and control of soft robots	*Nature*	^42^
2015 (3)	Wang and Iida	Deformation in soft-matter robotics: a categorization and quantitative characterization	*IEEE Robotics & Automation Magazine*	^28^
2015 (4)	Verl *et al.*	Soft robotics: transferring theory to application	*Germany: Springer-Verlag*	^77^
2015 (5)	Kruusamäe *et al.*	Self-sensing ionic polymer actuators: A review	*Actuators*	^78^
2016 (1)	Manti *et al.*	Stiffening in soft robotics: a review of the state of the art	*IEEE Robotics & Automation Magazine*	^79^
2016 (2)	Li *et al.*	Review of materials and structures in soft robotics	*Chinese Journal of Theoretical & Applied Mechanics*	^43^
2016 (3)	Laschi *et al.*	Soft robotics: technologies and systems pushing the boundaries of robot abilities	*Science Robotics*	^5^
2016 (4)	Aguilar *et al.*	A review on locomotion robophysics: the study of movement at the intersection of robotics, soft matter and dynamical systems	*Reports on Progress in Physics Physical Society*	^80^
2016 (5)	Hughes *et al.*	Soft manipulators and grippers: a review	*Frontiers in Robotics and AI*	^81^
2017 (1)	Lee *et al.*	Soft robot review	*International Journal of Control, Automation and Systems*	^60^
2017 (2)	Polygerinos *et al.*	Soft robotics: review of fluid-driven intrinsically soft devices; manufacturing, sensing, control, and applications in human-robot interaction	*Advanced Engineering Materials*	^66^
2017 (3)	Chen and Pei	Electronic muscles and skins: a review of soft sensors and actuators	*Chemical Reviews*	^82^
2017 (4)	Zhang *et al.*	Review of soft-bodied manipulator	*Journal of Mechanical Engineering*	^83^
2017 (5)	Wang *et al.*	Soft robotics: structure, actuation, sensing and control	*Journal of Mechanical Engineering*	^84^

**Table 10.** Technical Contents of Soft Robotics Related Reviews

*Item Year (No.)*	*Actuator*	*Fabrication*	*Control*	*Material*	*Sensing*	*Simulation*	*Bionics*	*Stiffness*	*Modeling*	*Power*	*Motion*	*Application*
1999	✓										✓	
2008 (1)	✓						✓					
2008 (2)	✓					✓			✓			✓
2009 (1)	✓	✓					✓					
2009 (2)	✓										✓	✓
2010	✓											✓
2011 (1)	✓		✓			✓	✓					
2011 (2)	✓	✓		✓								✓
2012 (1)	✓			✓	✓	✓	✓			✓		
2012 (2)	✓	✓					✓		✓			
2012 (3)	✓			✓	✓					✓		✓
2013	✓			✓			✓	✓				
2014 (1)	✓						✓	✓				
2014 (2)	✓	✓	✓				✓					
2014 (3)	✓			✓	✓		✓			✓		
2014 (4)	✓	✓		✓	✓					✓		
2015 (1)	✓			✓								
2015 (2)	✓	✓					✓		✓	✓	✓	
2015 (3)	✓			✓			✓			✓		✓
2015 (4)	✓	✓	✓	✓	✓	✓	✓		✓		✓	
2015 (5)	✓				✓				✓			
2016 (1)	✓			✓				✓		✓		
2016 (2)	✓	✓		✓			✓			✓	✓	✓
2016 (3)	✓	✓	✓		✓		✓	✓	✓		✓	✓
2016 (4)	✓			✓								✓
2016 (5)	✓			✓	✓							✓
2017 (1)	✓	✓	✓	✓	✓					✓	✓	
2017 (2)	✓	✓	✓		✓		✓			✓	✓	
2017 (3)	✓	✓			✓							✓
2017 (4)	✓	✓	✓	✓					✓			✓
2017 (5)	✓	✓	✓		✓							✓

Most of the reviews cover the latest work in this field around the world and summarize technical aspects as listed in Table 10. The reviewers always conclude with comments on the current problems and prospects for future work. These summaries, comments, and suggestions on techniques are important for fellow researchers and potential followers in this field. Thus, readers are encouraged to refer to the review documents listed in Table 9. In addition, these articles include discussions on new research directions and ideas that are expected to guide and stimulate creative work. Smart materials were mentioned in most reviews (referring to Table 10) and deeply discussed in most documents.^5,43,59,60^ Walker^59^ and Iida^28^ believe that collaboration between materials science and engineering will benefit soft robotics rather than isolated work. Bionics is also seen as a very inspiring source for soft robots, yet most articles caution that learning from biological form^61^ and motions^59,62,63^ is not the whole story. Biology-originated techniques or ideas, such as tissue engineering,^2,64^ camouflage,^65,66^ self-cleaning and self-healing,^5,65^ and even growth,^5^ are expected be integrated into the field to create life-like (or even living) soft robots. Another grand challenge for soft robot development is rapid virtual prototype techniques such as 3D printing,^59,67^ which require special modeling and simulation tools,^42,44,62^ totally different from those for rigid robots.^68^

Furthermore, some new advanced ideas were proposed, such as mechanical intelligence^64^ and task distribution^62^ for soft robot construction, modeling, and control. Pfeifer *et al.*^62^ and Iida and Laschi^44^ have advocated morphological computation and embodiment control for soft robot, which is also agreed by Rus and Tolley.^42^ Like conventional rigid robots, soft robot research started from social driving forces^63^ and it is expected to develop to commercial products.^2,42,62^ This will involve solving significant challenges of cost and safety,^28,64^ power supply,^28,43,62^ harvesting,^65,69^ and user and operator interfaces.^59^

Despite all of these aspects, the development of useful and sustainable soft robots will require advances toward a common fundamental theory that forms an infrastructural framework. This theoretical work requires both a deep knowledge of mathematics and a thorough understanding of soft robots. This is a grand challenge for the new discipline and it requires scientists and engineers with different scientific backgrounds, such as robotics, mechanical engineering, materials science, computer science, controls, chemistry, physics, biology, and mathematics. Furthermore, they are expected to cooperate intensively with each other to make substantive achievement in theoretical research.

## Conclusions

Although soft robotics is a relatively new field, there is no doubt that it is a rapidly growing topic in robotics. This is supported by the emerging publications, new related journals, and general public interest. The field is widely spreading and growing rapidly with institutions in the United States, Europe, and Asia contributing most of the original research, developing the infrastructure, and consolidating the academic community.

The growth of soft robotics requires new developments in complex structures, sensing, control, and power supplies that increasingly rely on advancing materials science and the corresponding manufacturing techniques. This is reflected in the number and prominence of articles in this field, discussing smart material applications and the appearance of materials science journals in the top list of soft robotics publications.

In reference to the journals, *IEEE Transactions on Robotics* ranks first among the top 20, whereas *Soft Robotics* holds the top position in journals categorized with “ROBOTICS” in the latest two consecutive years.

Whitesides, Wood, and Laschi are the top three most productive authors assessed by the ACPP and with highest h-index ranks. Furthermore, Whitesides research group occupies all the positions of top five authors with high total citations, which implies their powerful influence in the global soft robotics community. In addition to the top 10 authors, a large number of researchers are working in this field and they contributed 92.12% of the publications.

An analysis of reviews on soft robotics suggests that the following aspects are most attractive to researchers: actuators, fabrication, control, materials, sensing, simulation, bionics, stiffness, modeling, power, motion, and applications. Furthermore, multidisciplinary areas such as smart material, bionics, morphological computation, and embodiment control are expected to be major contributions to the field in the future. Some reviews discussed obstacles in the way of commercialization, but the development of a common fundamental theory for robot design and control seems to be a vital need for soft robotics to be an independent discipline.

## References

[1] Laschi C, Cianchetti M Soft robotics: new perspectives for robot bodyware and control. Front Bioeng Biotechnol 2014;2:3 25022259 10.3389/fbioe.2014.00003 PMC4090912

[2] Majidi C Soft robotics: a perspective—current trends and prospects for the future. Soft Robot 2014;1:5–11

[3] Tondu B, Lopez P Modeling and control of McKibben artificial muscle robot actuators. IEEE Control Syst 2000;20:15–38

[4] Albu-Schaffer A, Fischer M, Schreiber G, *et al.* Soft robotics: what Cartesian stiffness can obtain with passively compliant, uncoupled joints. IEEE/RSJ Int Conf Intell Robot Syst 2005;4:3295–3301

[5] Laschi C, Mazzolai B, Cianchetti M Soft robotics: technologies and systems pushing the boundaries of robot abilities. Sci Robot 2016;1:eaah3690 10.1126/scirobotics.aah3690 33157856

[6] Nickel VL, Perry J, Garrett AL, *et al.* Development of useful function in the severely paralyzed hand. J Bone Joint Surg 1963;45:933–952 14047365

[7] Krishna S, Nagarajan T, Rani AMA Review of current development of pneumatic artificial muscle. J Appl Sci 2011;11:1–7

[8] Shimachi S, Matumoto M A study on contact forces of soft fingers. Trans Jpn Soc Mech Eng C 1990;56:1440–1443

[9] Suzumori K, Tanaka H Flexible microactuator. J Jpn Soc Mech Eng 1991;94:600–602

[10] Suzumori K, Iikura S, Tanaka H Applying a flexible microactuator to robotic mechanisms. IEEE Control Syst 1992;12:21–27

[11] Suzumori K, Iikura S, Tanaka H Development of flexible microactuator and its applications to robotic mechanisms. IEEE International Conference on Robotics and Automation, 1991. Proc IEEE 1991;2:1622–1627

[12] Suzumori K, Maeda T, Wantabe H, *et al.* Fiberless flexible microactuator designed by finite-element method. IEEE ASME Trans Mechatron 1997;2:281–286

[13] Robinson G, Davies JBC The parallel bellows actuator. In Proceedings of Robotica 98, Brasov, Romania, 1998, pp. 195–200

[14] Kornbluh R, Pelrine R, Eckerle J, *et al.* Electrostrictive polymer artificial muscle actuators. IEEE International Conference on Robotics and Automation, 1998. Proc IEEE 2002;3:2147–2154

[15] Ozkan MOET, Inoue K, Negishi K, *et al.* Defining a neural network controller structure for a rubbertuator robot. Neural Netw 2000;13:533–544 10946398 10.1016/s0893-6080(00)00020-4

[16] Boblan I, Bannasch R, Schwenk H, *et al.* A human-like robot hand and arm with fluidic muscles: biologically inspired construction and functionality. In Proceedings of Ad-Hoc, Mobile, and Wireless Networks, Montreal, Canada, 2004, Vol. 3139, pp. 160–179

[17] Noritsugu T Development of pneumatic rotary soft actuator made of silicone rubber. J Robot Mechatron 2001;13:17–22

[18] Schulz S, Pylatiuk C, Bretthauer G A new ultralight anthropomorphic hand. IEEE International Conference on Robotics and Automation, 2001. Proc IEEE 2003;3:2437–2441

[19] Bao GJ, Yao PF, Xu ZG, *et al.* Pneumatic bio-soft robot module: structure, elongation and experiment. Int J Agric Biol Eng 2017;10:114–122

[20] Immega G, Antonelli K The KSI tentacle manipulator. IEEE International Conference on Robotics and Automation, 1995. Proc IEEE 2002;3:3149–3154

[21] Hannan MW, Walker ID The ‘elephant trunk’ manipulator, design and implementation. IEEE/ASME International Conference on Advanced Intelligent Mechatronics, 2001. Proc IEEE 2001;1:14–19

[22] Mcmahan W, Jones BA, Walker ID Design and implementation of a multi-section continuum robot: air-Octor. In Proceedings of IEEE/RSJ Intelligent Robots and Systems, Edmonton, Alberta, Canada, 2005, pp. 2578–2585

[23] Jones BA, Walker ID practical kinematics for real-time implementation of continuum robots. IEEE Trans Robot 2006;22:1087–1099

[24] Trimmer BA, Takesian AE, Sweet BM, *et al.* Caterpillar locomotion: a new model for soft-bodied climbing and burrowing robots. 7th International Symposium on Technology and the Mine Problem California, USA, 52006, pp. 1–10

[25] Chen G, Pham MT, Redarce T Sensor-based guidance control of a continuum robot for a semi-autonomous colonoscopy. Robot Auton Syst 2009;57:712–722

[26] Camarillo DB, Milne CF, Carlson CR, *et al.* Mechanics modeling of tendon-driven continuum manipulators. IEEE Trans Robot 2008;24:1262–1273

[27] Cianchetti M, Calisti M, Margheri L, *et al.* Bioinspired locomotion and grasping in water: the soft eight-arm OCTOPUS robot. Bioinspir Biomim 2015;10:035003 25970014 10.1088/1748-3190/10/3/035003

[28] Wang L, Iida F Deformation in soft-matter robotics: a categorization and quantitative characterization. Robot Autom Mag IEEE 2015;22:125–139

[29] Hernandez-Garcia YI, Chamizo JA, Kleiche-Dray M, *et al.* The scientific impact of mexican steroid research 1935–1965: a bibliometric and historiographic analysis. J Assoc Inf Sci Technol 2015;67:1245–1256

[30] Merigó JM, Rocafort A, Aznaralarcón JP Bibliometric overview of business & economics research. J Bus Econ Manag 2017;17:397–413

[31] Franceschet M A comparison of bibliometric indicators for computer science scholars and journals on Web of Science and Google Scholar. Scientometrics 2010;83:243–258

[32] Calma A, Davies M Academy of management journal, 1958–2014: a citation analysis. Scientometrics 2016;108:959–975

[33] Mingers J, Yang L Evaluating journal quality: a review of journal citation indicators and ranking in business and management. Eur J Oper Res 2016;257:323–337

[34] Heradio R, Torre LDL, Galan D, *et al.* Virtual and remote labs in education: a bibliometric analysis. Comput Educ 2016;98(C):14–38

[35] Chen H, Wan Y, Jiang S, *et al.* Alzheimer's disease research in the future: bibliometric analysis of cholinesterase inhibitors from 1993 to 2012. Scientometrics 2014;98:1865–1877

[36] Chen HQ, Wang X, He L, *et al.* Chinese energy and fuels research priorities and trend: a bibliometric analysis. Renew Sustain Energy Rev 2016;58:966–975

[37] Chen H, Qiu T, Rong JF, *et al.* Microalgal biofuel revisited: an informatics-based analysis of developments to date and future prospects. Appl Energy 2015;155:585–598

[38] Cundy TP, Sjd H, Marcus HJ, *et al.* Global trends in paediatric robot-assisted urological surgery: a bibliometric and progressive scholarly acceptance analysis. J Robot Surg 2018;12:109–115 28455800 10.1007/s11701-017-0703-3

[39] Laschi C, Mazzolai B, Mattoli V, *et al.* Design of a biomimetic robotic octopus arm. Bioinspir Biomim 2009;4:015006 19258690 10.1088/1748-3182/4/1/015006

[40] Laschi C, Rossiter J, Iida F, *et al.* Soft Robotics: Trends, Applications and Challenges. Livorno, Italy; Springer International Publishing, 2016

[41] Nakajima K, Li T, Kuppuswamy N, *et al.* How to harness the dynamics of soft body: timing based control of a simulated octopus arm via recurrent neural networks. Proc Comput Sci 2011;7:246–247

[42] Rus D, Tolley MT Design, fabrication and control of soft robots. Nature 2015;521:467–475 26017446 10.1038/nature14543

[43] Li T, Li G, Liang Y, *et al.* Review of materials and structures in soft robotics. Chin J Theor Appl Mech 2016;48:756–766

[44] Iida F, Laschi C Soft robotics: challenges and perspectives. Proc Comput Sci 2011;7:99–102

[45] Ilievski F, Mazzeo AD, Shepherd RF, *et al.* Soft robotics for chemists. Angewandte Chemie 2011;50:1890–1895 21328664 10.1002/anie.201006464

[46] Yang GZ, Bellingham J, Choset H, *et al.* Science for robotics and robotics for science. Sci Robot 2016;1:eaal2099 10.1126/scirobotics.aal2099 33157859

[47] Tahamtan I, Afshar AS, Ahamdzadeh K Factors affecting number of citations: a comprehensive review of the literature. Scientometrics 2016;107:1195–1225

[48] Pfeifer R, Lungarella M, Iida F Self-organization, embodiment, and biologically inspired robotics. Science 2007;318:1088–1093 18006736 10.1126/science.1145803

[49] Shepherd RF, Ilievski F, Choi W, *et al.* Multigait soft robot. Proc Natl Acad Sci U S A 2011;108:20400 22123978 10.1073/pnas.1116564108 PMC3251082

[50] Tee BC, Wang C, Allen R, *et al.* An electrically and mechanically self-healing composite with pressure- and flexion-sensitive properties for electronic skin applications. Nat Nanotechnol 2012;7:825–832 23142944 10.1038/nnano.2012.192

[51] Kim Y, Jian Z, Yeom B, *et al.* Stretchable nanoparticle conductors with self-organized conductive pathways. Nature 2013;500:59–63 23863931 10.1038/nature12401

[52] Chen Z, Shatara S, Tan X Modeling of biomimetic robotic fish propelled by an ionic polymer–metal composite caudal fin. IEEE ASME Trans Mechatron 2010;15:448–459

[53] Kim S, Spenko M, Trujillo S, *et al.* Smooth vertical surface climbing with directional adhesion. IEEE Trans Robot 2008;24:65–74

[54] Suo Z Theory of dielectric elastomers. Acta Mech Solida Sin 2010;23:549–578

[55] Amjadi M, Kyung K, Park I, *et al.* Stretchable, skin-mountable, and wearable strain sensors and their potential applications: a review. Adv Funct Mater 2016;26:1678–1698

[56] Polygerinos P, Wang Z, Galloway KC, *et al.* Soft robotic glove for combined assistance and at-home rehabilitation. Robot Auton Syst 2015;73(C):135–143

[57] Park SJ, Gazzola M, Park KS, *et al.* Phototactic guidance of a tissue-engineered soft-robotic ray. Science 2016;353:158–162 27387948 10.1126/science.aaf4292 PMC5526330

[58] Robinson G, Davies JBC Continuum robots—a state of the art. IEEE International Conference on Robotics and Automation, 1999. Proc IEEE 2002;4:2849–2854

[59] Trivedi D, Rahn CD, Kier WM, *et al.* Soft robotics: biological inspiration, state of the art, and future research. Appl Bionics Biomech 2008;5:99–117

[60] Lee C, Kim M, Kim YJ, *et al.* Soft robot review. Int J Control Autom Syst 2017;15:3–15

[61] Wang Y, Yang X, Chen Y, *et al.* A biorobotic adhesive disc for underwater hitchhiking inspired by the remora suckerfish. Sci Robot 2017;2:1–9 10.1126/scirobotics.aan8072 33157888

[62] Pfeifer R, Lungarella M, Iida F The challenges ahead for bio-inspired ‘soft’ robotics. Commun ACM 2012;55:76–87

[63] Bahramzadeh Y, Shahinpoor M A review of ionic polymeric soft actuators and sensors. Soft Robot 2014;1:38–52

[64] Kim S, Laschi C, Trimmer B Soft robotics: a bioinspired evolution in robotics. Trends Biotechnol 2013;31:287–294 23582470 10.1016/j.tibtech.2013.03.002

[65] Bauer S, Bauer-Gogonea S, Graz I, *et al.* 25th anniversary article: a soft future: from robots and sensor skin to energy harvesters. Adv Mater 2014;26:149–161 24307641 10.1002/adma.201303349 PMC4240516

[66] Polygerinos P, Correll N, Morin SA, *et al.* Soft robotics: review of fluid-driven intrinsically soft devices; manufacturing, sensing, control, and applications in human-robot interaction. Adv Eng Mater 2017;19:1700016

[67] Tan DP, Ji SM, Fu YZ An improved soft abrasive flow finishing method based on fluid collision theory. Int J Adv Manuf Technol 2016;85:1261–1274

[68] Tan DP, Ji SM, Jin MS Intelligent Computer-Aided Instruction Modeling and a Method to Optimize Study Strategies for Parallel Robot Instruction. IEEE Trans Educ 2013;56:268–273

[69] Chen JL, Yang JX, Zhao J, *et al.* Energy demand forecasting of the greenhouses using nonlinear models based on model optimized prediction method. Neurocomputing 2016;174(PB):1087–1100

[70] Zhang LB, Bao GJ, Yang QH, *et al.* Review on flexible pneumatic actuator and its application in dexterous hand. Chin Mech Eng 2008;19:2891–2897

[71] Cho KJ, Koh JS, Kim S, *et al.* Review of manufacturing processes for soft biomimetic robots. Int J Precis Eng Manuf 2009;10:171–181

[72] Greef AD, Lambert P, Delchambre A Towards flexible medical instruments: review of flexible fluidic actuators. Precis Eng 2009;33:311–321

[73] De Volder M, Reynaerts D Pneumatic and hydraulic microactuators: a review. J Micromech Microeng 2010;20:043001

[74] Cao YJ, Shang JZ, Liang KS, *et al.* Review of soft-bodied robots. J Mech Eng 2012;48:25–33

[75] Otero TF, Martinez JG, Arias-Pardilla J Biomimetic electrochemistry from conducting polymers. A review: artificial muscles, smart membranes, smart drug delivery and computer/neuron interfaces. Electrochim Acta 2012;84:112–128

[76] Elango N, Faudzi AAM A review article: investigations on soft materials for soft robot manipulations. Int J Adv Manuf Technol 2015;80:1027–1037

[77] Verl A, Albu-Schäffer A, Brock O, *et al.* Soft robotics: transferring theory to application. Berlin, Germany; Springer Publishing Company, Incorporated, 2015:141–148

[78] Kruusamäe K, Punning A, Aabloo A, *et al.* Self-sensing ionic polymer actuators: a review. Actuators 2015;4:17–38

[79] Manti M, Cacucciolo V, Cianchetti M Stiffening in soft robotics: a review of the state of the art. IEEE Robot Autom Mag 2016;23:93–106

[80] Aguilar J, Zhang T, Qian F, *et al.* A review on locomotion robophysics: the study of movement at the intersection of robotics, soft matter and dynamical systems. Rep Prog Phys 2016;79:110001 27652614 10.1088/0034-4885/79/11/110001

[81] Hughes J, Culha U, Giardina F, *et al.* Soft manipulators and grippers: a review. Front Robot AI 2016;3:1–12

[82] Chen D, Pei Q Electronic muscles and skins: a review of soft sensors and actuators. Chem Rev 2017;117:11239–11268 28816043 10.1021/acs.chemrev.7b00019

[83] Zhang JH, Wang T, Hong J, *et al.* Review of software manipulator. J Mech Eng 2017;53:19–28

[84] Wang TM, Hao YF, Yang XB, *et al.* Soft robotics: structure, actuation, sensing and control. J Mech Eng 2017;53:1–13

